# Should we be treating animal schistosomiasis in Africa? The need for a One Health economic evaluation of schistosomiasis control in people and their livestock

**DOI:** 10.1093/trstmh/trx047

**Published:** 2017-10-16

**Authors:** Charlotte M Gower, Louise Vince, Joanne P Webster

**Affiliations:** a Centre for Emerging, Endemic and Exotic Diseases (CEEED), Pathobiology and Population Sciences, Royal Veterinary College, University of London, London AL9 7TA, UK; b London Centre for Neglected Tropical Disease Research (LCNTDR), Faculty of Medicine, Imperial College London, Norfolk Place, London W2 1PG, UK

**Keywords:** Animals, Economic evaluation, Humans, One Health, Praziquantel, Schistosomiasis

## Abstract

A One Health economic perspective allows informed decisions to be made regarding control priorities and/or implementation strategies for infectious diseases. Schistosomiasis is a major and highly resilient disease of both humans and livestock. The zoonotic component of transmission in sub-Saharan Africa appears to be more significant than previously assumed, and may thereby affect the recently revised WHO vision to eliminate schistosomiasis as a public health problem by 2025. Moreover, animal schistosomiasis is likely to be a significant cost to affected communities due to its direct and indirect impact on livelihoods. We argue here for a comprehensive evaluation of the economic burden of livestock and zoonotic schistosomiasis in sub-Saharan Africa in order to determine if extending treatment to include animal hosts in a One Health approach is economically, as well as epidemiologically, desirable.

## Introduction

The One Health approach recognizes that the health of humans is connected to the health of animals and the environment, and aims to encourage collaborative effects of multiple agencies to achieve the best outcomes for each.^1^ A One Health economic perspective that can accommodate all costs and benefits to human and livestock sectors, as well as consideration of where an integration of service delivery itself may have additional cost-savings or benefits, allows informed decisions to be made about control priorities and implementation strategies for infectious diseases. For example, the proposed control of brucellosis in Mongolia, through the vaccination of 25 million cattle, sheep and goats at a cost of US$8 million, was initially considered economically unjustifiable, based on the estimated US$3 million costs of zoonotic infections to public health services alone. However, the programme was subsequently deemed cost-effective due to the total estimated benefit of US$26 million when productivity losses to the human and agricultural sector were included within a One Health economic evaluation.^2^

### Schistosomiasis and its control in Africa

Human schistosomiasis is a chronic and debilitating neglected tropical disease (NTD) that infects over 140 million people, with over 90% of cases occurring in sub-Saharan Africa.^3^ Livestock schistosomiasis infections due to *Schistosoma bovis, Schistosoma curassoni* and/or *Schistosoma matthei* in cattle, sheep and goats are also prevalent in sub-Saharan Africa, and are likely to be a highly important, if overlooked, cause of animal mortality and morbidity.^4^*S. mansoni*, a causative agent of human intestinal schistosomiasis in Africa, is acknowledged to be potentially zoonotic, with reservoirs in both rodents and primates.^4^ In contrast, *Schistosoma haematobium*, the causative agent of urogenital schistosomiasis in Africa, was originally believed to be an exclusively human-specific parasite. However, morphological and subsequent molecular studies of parasites isolated from, for example, children in West Africa have identified viable hybrids of human *S. haematobium* with livestock *S. bovis* and/or *S. curassoni*,^5^ as well as between *S. bovis* with *S. curassoni* alone,^6^ demonstrating that there is clearly a zoonotic component of transmission (see Leger and Webster^7^ for a full review of zoonotic hybrid species identification across sub-Saharan African countries).

Current control of human schistosomiasis in Africa is based on preventative chemotherapy (PC), whereby populations are mass treated with the donated antihelminthic, praziquantel (PZQ). These programmes, in general, have had impressive effects on reducing human helminthic infection prevalence, intensity and associated morbidity,^8^ to the extent that a shift towards interruption of transmission has been argued for in some cases.^9^ Schistosome control programmes in South and South-East Asia, where schistosomiasis is caused by the highly zoonotic *Schistosoma japonicum* and/or *Schistosoma mekongi*, have involved mass treatment of both humans and bovines with PZQ, as well as essential improvements to water, sanitation and health education, replacement of bovines with tractors, and in some areas attempts to develop a bovine vaccine.^10^ Whilst, within certain regions of sub-Saharan Africa, some progress in improvements to water, sanitation and health education has been made, there are no such formal antischistosome control or donation programmes involving animals, although there are undocumented reports and observations of informal/unstructured treatment of animals with (locally purchased) PZQ for agricultural purposes in West Africa (all authors, personal observations), on an as-yet unquantified scale.

Concurrent treatment of zoonotic *Schistosoma* spp. reservoirs, at least in terms of livestock hosts in sub-Saharan Africa (rodent control remains a global challenge), is likely to be imperative for successful transmission interruption of human disease.^8^ However, a key problem for treatment of many zoonotic infections with livestock reservoirs is that, while the costs of treatment fall largely on the agricultural sector, the benefits of reduced transmission to humans are felt largely by the public health and medical sectors. Therefore, motivating the sustainable involvement of livestock authorities and producers, who may have other disease priorities, can often prove difficult.^11^ Economic arguments can, however, provide a strong justification for local and national governments to act. Given the potential impact of schistosomiasis on animal health and productivity, a One Health economic evaluation of extending treatment to animal hosts in sub-Saharan Africa appears warranted, and requires data to be gathered on the costs and benefits to both sectors. With a focus on economic cost aspects, we briefly consider here the available information and highlight current gaps in knowledge.

### Cost-effectiveness of preventative chemotherapy in humans

Preventative chemotherapy is highly cost effective, with, for example, recent estimates of the cost effectiveness of treating schoolchildren in Côte d'Ivoire against schistosomiasis of US$118 per disability adjusted life years (DALY) averted, as compared with no-treatment controls.^12^ Further analyses have shown that increasing coverage to include the wider community, and adults in particular, is also highly cost effective, with an incremental cost-effectiveness ratio of $167 per DALY averted as compared with treating schoolchildren alone.^12^ Integration with other human-focused NTD control programmes, such as those for soil-transmitted helminthiasis, onchocerciasis and lymphatic filariasis, has led to further cost savings within many countries across sub-Saharan Africa.^8,13^ Costs vary, of course, depending on the prevalence of infection and are likely to increase in areas of low prevalence, but recent work suggests that mass treatment with PZQ is cost effective at 5% in children or 15% in whole communities, prevalences that are below current WHO treatment guidelines.^14^ Estimates of cost-effectiveness in such studies are based on the 10 clinical outcomes of schistosomiasis included in the Global Burden of Disease studies. There is also evidence of productivity losses in human populations due to poor school attendance, poorer educational outcomes and reduced worker productivity, such as through lost work days,^15,16^ as well as costs to tourists of acute schistosomiasis,^17^ all of which have not been assessed in current economic evaluations, suggesting that the cost-effectiveness of human treatment may be even higher than currently reported.

### Cost-effectiveness of treatment of animal and zoonotic schistosomiasis

The economic importance of schistosomiasis to livestock farmers is suspected to be high, although there are substantially fewer data available on the economic impact of schistosomiasis in animals or the cost-effectiveness of their treatment. Helminth infections of ruminants are widely acknowledged as a constraint on efficient livestock production systems globally^18^ and there is some literature available documenting the pathological effects of schistosome infection in animals, such as of *S. mattheei* in cattle,^19^*S. bovis* in goats^20^ and *S. curassoni* in sheep.^21^ Productivity losses may include mortality of infected animals, growth delay, reduced meat and milk quantity and quality, and poor future reproductive capacity.^22^ In Sudan, *S. bovis* mortality of 6–18 month old cattle was estimated at 7.1%^23^ and prevalences as high as 90% were reported. There are even fewer data available on the potential costs of treatment, including current spending by farmers on PZQ treatment, where wide-scale treatment programmes would have additional costs, such as the strengthening of veterinary services. Nevertheless, one economic evaluation of human disease control in China, which did incorporate costs of also treating bovines, reported a cost–benefit ratio of US$6.20 for every dollar spent.^24^ In the only (to the authors’ knowledge) economic evaluation of control of an animal schistosome in Africa, through proposed vaccination of cattle in Sudan, returns ranging from US$0.7 to 12.7 per dollar spent were estimated depending on parasite prevalence and the costs of developing an effective vaccine,^22^ although this has not yet been achieved over 30 years later. There are, furthermore, very few data even on the prevalence and intensity of animal schistosomiasis in sub-Saharan Africa, or on the relative importance of zoonotic transmission cycles to human disease, either in the past nor under current environments under extensive levels of anthropogenic change.^4^

### Potential costs and caveats of a One Health Approach

The use of the same drugs in animals and humans raises profound issues regarding the potential selection of PZQ resistance. Veterinary use and misuse of antihelminthics increases the risk of drug resistance evolving, particularly since common veterinary schedules for antihelminthic treatment are often more frequent than those used in annual human PC. Likewise, appropriate veterinary dosages are often not as well studied or always implemented within developing country settings. Furthermore, untreated zoonotic reservoirs may currently be acting as a *refugia*, slowing the development of drug resistance.^4^ Co-treatment of livestock with PZQ could thus represent both an immediate cost to human treatment programmes, if treatment fails, and a negative externality at the societal level, due to the reduced effectiveness of the developed drug.^25^ It is therefore imperative that any extension of PZQ treatment to livestock hosts be accompanied by the accessibility and implementation of appropriate veterinary PZQ formulations and that all treatment programmes must incorporate comprehensive monitoring and evaluation within the livestock host, particularly in terms of assessing ongoing drug efficacies, to ensure that human disease control successes are protected.

### Conclusions and recommendations

We urgently need reliable estimates of the current prevalence, intensity and pathological burden of animal schistosomes in affected communities in sub-Saharan Africa concurrently incorporating these data within a One Health economic evaluation of extending disease control activities to animal hosts. Costs and benefits are incurred in both private and public sectors for both human health and livestock production and, therefore, we need analyses that include human and livestock health and production from a societal perspective. Methods now exist for the cost assignment of treating zoonotic reservoirs to protect human health in terms of DALY averted, which provide motivation to public health authorities.^2^ More widespread treatment of animal schistosomes, if seen to be medically and economically advantageous to these communities, as well as the separate, but important potential animal welfare considerations, will require improvements in animal, as well as human health education, strengthening of veterinary services, accessible and affordable veterinary formulations of PZQ (for which the authors have already observed a demand), integrally combined with risk assessment and careful monitoring of both drug supply and the potential development of drug resistance throughout. Above all, this will require political will and endorsement, across both the international and regional agendas, as well as national and local stability. It should be remembered that schistosomiasis, like so many coinfecting NTDs within these communities, is a dynamic and resilient disease impacting the rural poor, where in many cases it is the same families being affected by the disease in themselves, their children and their animals, such that costs and benefits are deeply intertwined. In the Anthropocene epoch where multilateral organizations often set the agenda for the poorest of the world, we have a social responsibility to evaluate how this disease affects all actors and respond accordingly and appropriately.

## References

[1] Centers for Disease Control and Prevention (https://www.cdc.gov/onehealth/) (accessed 24 August 2017).

[2] Zinsstag J, Choudhury AA, Roth F et al One Health economics In: Zinsstag J (ed.) One Health: the theory and practice of integrated health approaches. Wallingford:CABI; 2015, p. 114.

[3] Colley DG, Bustinduy AL, Secor WE et al Human schistosomiasis. Lancet 2014;383:2253–64. 24698483 10.1016/S0140-6736(13)61949-2 PMC4672382

[4] Webster JP, Gower CM, Knowles SC et al One Health—an ecological and evolutionary framework for tackling neglected zoonotic diseases. Evol Appl 2016;9:313–33. 26834828 10.1111/eva.12341 PMC4721077

[5] Webster BL, Diaw OT, Seye M et al Introgressive hybridization of *Schistosoma haematobium* group species in Senegal: species barrier break down between ruminant and human schistosomes. PLoS Negl Trop Dis 2013; 7:e2110. 23593513 10.1371/journal.pntd.0002110 PMC3617179

[6] Leger E, Garba A, Hamidou AA et al Introgressed animal schistosomes *Schistosoma curassoni* and *S. bovis* naturally infecting humans. Emerg Infect Dis 2016;22 :2212–4. 27869609 10.3201/eid2212.160644 PMC5189150

[7] Leger E, Webster JP *Schistosoma* spp. hybridizations: implications for evolution, epidemiology and control. Parasitology 2017;144:65–80 27572906 10.1017/S0031182016001190

[8] Webster JP, Molyneux DH, Hotez PJ et al The contribution of mass drug administration to global health: past, present and future. Philos Trans R Soc Lond B Biol Sci 2014;369:20130434. 24821920 10.1098/rstb.2013.0434 PMC4024227

[9] Tchuem Tchuente LA, Rollinson D et al Moving from control to elimination of schistosomiasis in sub-Saharan Africa: time to change and adapt strategies. Infect Dis Poverty 2017;6:42. 28219412 10.1186/s40249-017-0256-8 PMC5319063

[10] Cao ZG, Zhao YE, Lee Willingham A et al Towards the elimination of *Schistosomiasis japonica* through control of the disease in domestic animals in the People's Republic of China: a tale of over 60 years. Adv Parasitol 2016;92:269–306. 27137450 10.1016/bs.apar.2016.03.001

[11] Shaw A The economics of zoonoses and their control In: Rushton J (ed.) The economics of animal health and production. Wallingford: CABI; 2012, pp. 161–7.

[12] Lo NC, Bogoch II, Blackburn BG et al Comparison of community-wide, integrated mass drug administration strategies for schistosomiasis and soil-transmitted helminthiasis: a cost-effectiveness modelling study. Lancet Glob Health 2015;3:e629–38. 26385302 10.1016/S2214-109X(15)00047-9

[13] Leslie J, Garba A, Boubacar K et al Neglected tropical diseases: comparison of the costs of integrated and vertical preventive chemotherapy treatment in Niger. Int Health 2013;5:78–84. 24029850 10.1093/inthealth/ihs010

[14] Lo NC, Lai YS, Karagiannis-Voules DA et al Assessment of global guidelines for preventive chemotherapy against schistosomiasis and soil-transmitted helminthiasis: a cost-effectiveness modelling study. Lancet Infect Dis 2016;16:1065–75. 27286968 10.1016/S1473-3099(16)30073-1

[15] King CH, Dangerfield-Cha M The unacknowledged impact of chronic schistosomiasis. Chronic Illn 2008;4 :65–79. 18322031 10.1177/1742395307084407

[16] Lenk EJ, Redekop WK, Luyendijk M, et al Productivity loss related to neglected tropical diseases eligible for preventive chemotherapy: a systematic literature review. PLoS Negl Trop Dis 2016;10(2):e0004397. 26890487 10.1371/journal.pntd.0004397 PMC4758606

[17] Leshem E, Maor Y, Meltzer E et. al Acute schistosomiasis outbreak: clinical features and economic impact. Clin Infect Dis 2008;47:1499–506. 18990059 10.1086/593191

[18] Charlier J, Velde FV, van der Voort M et al ECONOHEALTH: placing helminth infections of livestock in an economic and social context. Vet Parasitol 2015;212:62–7. 26159836 10.1016/j.vetpar.2015.06.018

[19] De Bont J, Shaw DJ, Vercruysse J The relationship between faecal egg counts, worm burden and tissue egg counts in early *Schistosoma mattheei* infections in cattle. Acta Trop 2002;81:63–76. 11755433 10.1016/s0001-706x(01)00198-x

[20] Kassuku A, Christensen NO, Nansen P et al Clinical pathology of *Schistosoma bovis* infection in goats. Res Vet Sci 1986;40:44–7. 2871603

[21] Vercruysse J, Southgate VR, Rollinson D The epidemiology of human and animal schistosomiasis in the Senegal River Basin. Acta Trop 1985;42:249–59. 2865881

[22] McCauley EH Economic evaulation of the production impact of bovine schistsosomiaisis and vaccination in the Sudan. Prev Vet Med 1984;2:735–54.

[23] McCauley EH, Tayeb A, Majid AA Owner survey of schistosomiasis mortality in Sudanese cattle. Trop Anim Health Prod 1983;15:227–33. 6649061 10.1007/BF02242065

[24] Zhou XN, Wang LY, Chen MG et al An economic evaluation of the national schistosomiasis control programme in China from 1992 to 2000. Acta Trop 2005;96:255–265. 16154104 10.1016/j.actatropica.2005.07.026

[25] Oppong R, Smith RD, Little P et al Cost effectiveness of amoxicillin for lower respiratory tract infections in primary care: an economic evaluation accounting for the cost of antimicrobial resistance. Br J Gen Pract 2016;66:e633–9. 27402969 10.3399/bjgp16X686533 PMC5198702

